# Return to Sport and Performance After Thumb Metacarpophalangeal Joint Collateral Ligament Surgery in the National Basketball Association

**DOI:** 10.7759/cureus.42499

**Published:** 2023-07-26

**Authors:** Brendan M Holderread, Jordan Jafarnia, Brian Phelps, Mark Perrin, Robert A Jack, Joshua D Harris, Shari R Liberman

**Affiliations:** 1 Orthopedics & Sports Medicine, Houston Methodist Hospital, Houston, USA

**Keywords:** return to sport, professional athlete, national basketball association, basketball, athlete, metacarpophalangeal joint, thumb, radial collateral ligament, ulnar collateral ligament, collateral ligament

## Abstract

Introduction

Basketball players are at increased risk of thumb collateral ligament injury (ulnar collateral ligament (UCL) and radial collateral ligament (RCL)).

Methods

The National Basketball Association (NBA) players with thumb collateral ligament surgery were identified using publicly available data. Performance statistics, ligament injuries (UCL or RCL), return to sport (RTS) time, laterality, and injury dates were recorded. Cases were matched 1:1 with controls based on age (±1 year), body mass index (BMI), NBA experience (±1 year), and performance statistics prior to the index date. RTS was defined as playing in one NBA game postoperatively. Career longevity was evaluated. Summary statistics were calculated, and Student's t-tests (ɑ = 0.001) were performed.

Results

All 47 players identified with thumb collateral ligament surgeries returned to sport. Thirty-three players (age: 26.9 ± 3.0) had one year of postoperative NBA experience for performance analysis. Career length (case: 9.6 ± 4.1, control: 9.4 ± 4.3, p > 0.001) was not significantly different from controls (p > 0.001). The same season time to RTS (n = 20) was 7.1 ± 2.4 weeks. Off-season or season-ending surgery (n = 13) RTS time was 28.4 ± 18.7 weeks. Neither thumb collateral ligament (UCL, n = 7; RCL, n = 10; unknown, n = 16) had an identifiable difference between the groups when evaluating career length. Career length, games/season, and performance were not different for players who underwent surgery on their dominant thumb (63.6%, 21/33) compared to controls (p > 0.001).

Conclusion

RTS rate is high in NBA athletes undergoing thumb collateral ligament surgery. Players do not experience decreased performance or career length due to thumb collateral ligament surgery, regardless of a dominant or non-dominant thumb injury.

## Introduction

The National Basketball Association (NBA) is the world’s most recognized professional basketball league [1]. Since the league’s inception in 1946, NBA basketball has progressively developed into a physical game in which contact is prevalent [2]. Increased contact creates an opportunity for NBA players to suffer a variety of musculoskeletal injuries.

The upper extremity is involved in 19.3% of all game-related injuries in the NBA [3]. Specifically, injuries to the thumb represent 2.7% of all game-related injuries [3]. Ligamentous thumb injuries are commonly characterized as ulnar collateral ligament (UCL) or radial collateral ligament (RCL) sprain or tears [4]. Thumb collateral ligament injuries occur from a variety of mechanisms, including catching a ball, attempted forceful grasping, or falling on an abducted thumb [5]. Thumb collateral ligaments are the most common injuries to the thumb. Their (RCL and UCL) respective mechanisms of injury to the thumb collateral ligaments are (1) a radially directed force on an extended thumb leading to stretch and rupture of the UCL in addition to accessory stabilizers of the metacarpophalangeal (MCP) joint and (2) a similar mechanism, but with an ulnar directed force that will injure the RCL [6].

Both UCL and RCL injuries are graded from 1 to 3, with grades 1 and 2 considered incomplete tears that can first be managed non-operatively. A key physical examination finding to differentiate a complete from an incomplete tear is the lack of a firm endpoint when applying varus/valgus stress to test the ligaments. Treatment options for grade 3 thumb collateral ligament injury depend on the amount of varus/valgus instability, specific to the UCL, and the presence of a Stener lesion. A Stener lesion is when the distal aspect of the UCL insertion avulses and becomes separated from its insertion on the proximal phalanx by the adductor pollicis aponeurosis. In acute cases of complete tears involving the UCL or RCL in high-level athletes, surgical repair or reconstruction should be considered to restore MCP joint stability and pinch strength, prevent the development of osteoarthritis (OA), and return to sport (RTS) [4,6]. RTS clearance depends on repair/reconstruction healing, so athletes may withstand contact stresses to the joint. Previous literature has demonstrated an RTS time of 9.6 weeks after surgically repaired thumb ligament tears in NBA basketball players, but the impact on performance has not been evaluated [7].

The purpose of this study was to identify NBA basketball players who underwent thumb collateral ligament surgery (UCL or RCL) to evaluate (1) the time to RTS and (2) post-injury career length compared to matched controls, (3) compare preoperative performance to postoperative performance in identified players, and (4) compare post-injury performance to matched controls. The authors hypothesized NBA players who underwent thumb ligament repair surgery would have (1) an RTS rate of >90%, (2) no significant difference in the postoperative career length, (3) no significant differences in their preoperative and postoperative performance, and (4) no significant difference in performance when compared to matched control players.

This article was previously presented as a meeting abstract at the 2023 AAHS Annual Meeting on January 19, 2023.

## Materials and methods

This study utilized publicly available information; therefore, Institutional Review Board approval was not sought. All data were collected between September 21, 2021, and December 1, 2021.

**Figure 1:**
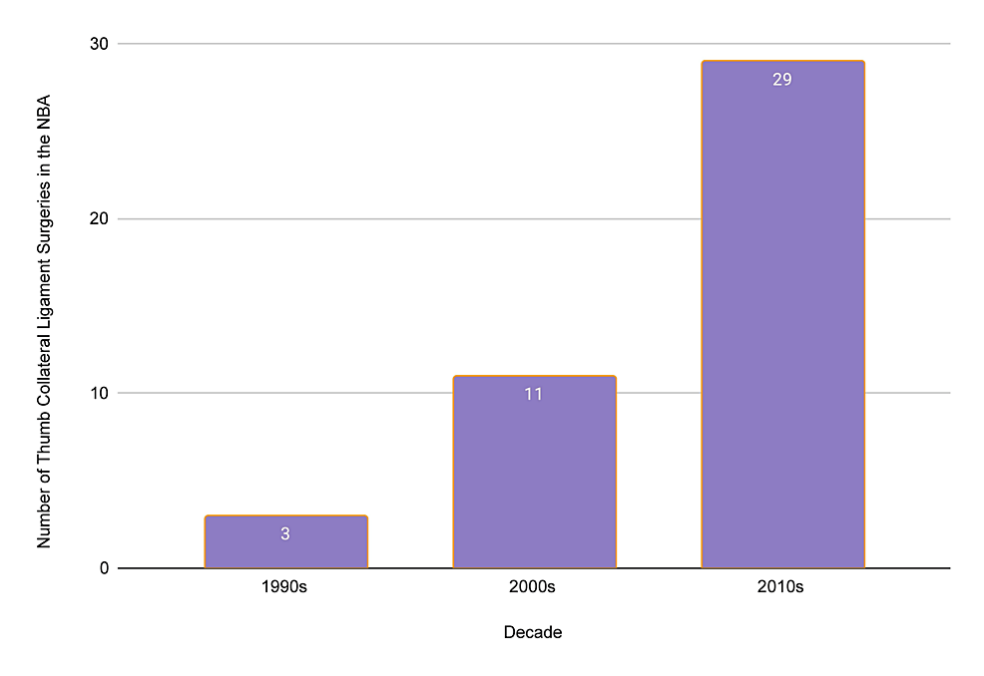
Thumb collateral ligament surgeries in National Basketball Association (NBA) players, decade to decade * Four players underwent surgery in the year 2020 or 2021. ** Thirty-three players were included in the return to sport analysis.

**Figure 2:**
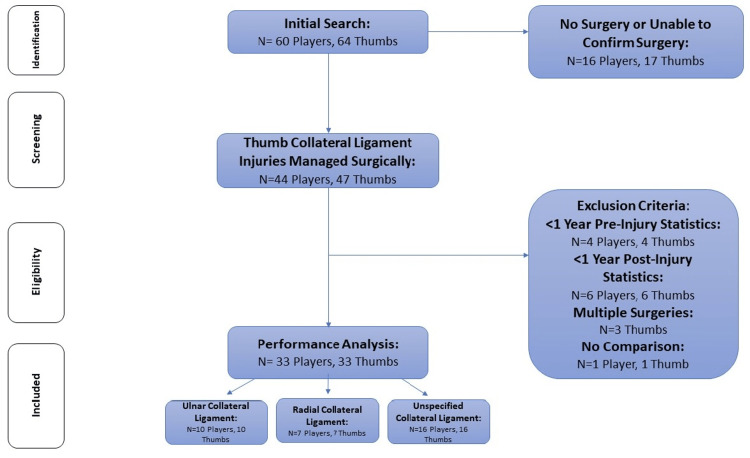
Flowchart demonstrating search strategy and selection of players for performance analysis

A manual, systematic search performed queried a combination of an NBA team’s name with “thumb ligament” (example: “Houston Rockets Thumb Ligament”). This was repeated for every current NBA team using a list from the official NBA webpage. Similar search strategies and methodology have been utilized in prior RTS evaluations after injury in other professional sports, including the NBA [4,8-13]. NBA players who injured a thumb collateral ligament between 1992 and 2021 were identified through NBA team websites, web-based injury reports, and team press releases (Figures 1, 2).

Only cases with confirmed thumb collateral ligament surgery by at least one source were included for data analysis. No methodology or public record was available to determine collateral ligament repair versus reconstruction. No reliable method for identifying Stener lesions in thumb UCL was identified. No reliable method for identifying simultaneous RCL/UCL tears was identified. For each date of surgery, the season in which surgery occurred was defined as the "index season." For example, a player with 15 years of career experience who was injured in season three of their career would have two seasons of "pre-index" performance statistics with 12 years of post-index experience.

The following exclusion criteria were applied for performance analysis: players who received non-operative management, unable to confirm thumb collateral ligament surgery, players with less than one year of pre-index season or post-index season experience/performance statistics, revision/multiple thumb collateral ligament surgeries, and players who did not have an acceptable control for comparison. All four players with less than a year of pre-index season experience and performance statistics were rookies. Three players with less than a year of post-index season experience and performance statistics returned to play internationally or in the G-League (the official minor league organization of the NBA) and the remaining three were injured in the year 2021 when the investigation was performed.

A control group was selected based on age (±1 year), BMI, NBA experience (±1 year), and performance statistics prior to the index date. Positions were utilized for matching, but rule changes in the NBA have led teams to favor "positionless" basketball. Information about positions was not recorded for the outcome reporting for this reason [13]. Selected control players were assigned a matching index date with the case player for comparison. A Kaplan-Meier career survival curve used a player's retirement as the endpoint.

Recorded variables included BMI, dominant hand (hand utilized for perimeter jump shooting), laterality of surgery, injured collateral ligament (thumb UCL, thumb RCL, or unspecified ligament), injury setting, in-season injury, season-ending (due to late season injury) or off-season surgery, date of surgery, date of RTS, and performance statistics.

Performance statistics were recorded as career totals and collected in relation to the index season (pre-index season and post-index season). Performance statistics recorded include the number of seasons, games played, games started, minutes played, made field goals (FG), FG attempts, FG %, three-point shots made, three-point shots attempted, three-point shooting %, two-point shots made, two-point shots attempted, two-point shooting %, effective FG %, free throws (FT) made, FT attempted, FT %, offensive rebounds/game, defensive rebounds/game, total rebounds/game, assists per game, steals per game, blocks per game, turnovers per game, personal fouls per game, points per game, and league adjusted shooting statistics.

League-adjusted shooting statistics normalize a player's shooting performance based on league averages for the metric of interest. The league adjustment provides a control for the varying pace of play in the NBA over time, which may impact performance statistics that are based on totals. League-adjusted statistics recorded include FG+ (field goal+), 2P+ (two-point shooting+), 3P+ (three-point shooting+), eFG+ (effective field goal percentage+), FT+ (free throw percentage+), TS+ (true shooting percentage+), FTr+ (free throw attempt rate+), and 3PAr+ (three-point attempt rate+). These statistics are centered around the number 100, which represents the league average. For example, a 3PAr+ of 110 represents a player who attempted three-point shots at a rate 10% higher than the league average in the same time period.

Summary statistics were aggregated and calculated for all collected variables and performance statistics. Comparisons were evaluated for significant differences with two-tailed paired sample Student's t-tests with ɑ = 0.001 (p < 0.001 was significant). The present study defined RTS as returning to play in at least one professional basketball game. Participation in a league other than the NBA was noted. Time to RTS was calculated for thumb collateral ligament surgeries that met inclusion criteria for performance analysis (n = 33). A Kaplan-Meier survivorship curve was generated to analyze postoperative career length between the case and control player. Retirement was set as the end of a player's career.

## Results

Forty-nine players were identified by the search strategy with an identifiable thumb collateral ligament injury. Forty-seven players (47/49, 95.9%) with thumb collateral ligament injuries who went on to have surgery (44 primaries, three revisions) were identified as undergoing thumb collateral ligament surgery. All forty-seven players with thumb collateral ligament surgeries returned to play either in the NBA, an NBA professional affiliate (G-League, formerly known as NBA developmental league), or professional basketball in another league. After applying the exclusion criteria for performance analysis, 33 thumb collateral ligament surgeries were available. Seventeen of those injuries occurred during regular season games (51.5%). No significant differences existed between the case group and the control group when evaluating the player’s age at the time of surgery, BMI, and pre-injury seasons in NBA (p > 0.001) (Table 1).

**Table 1:** Case and control characteristics utilized for matching and injury setting for included thumb collateral ligament surgeries of case players * Statistics presented as mean ± SD or n (%).

Comparison of case and control groups		Case	Control	p-value
	Number of players	33	33	
	Age at surgery	26.9 ± 3.0	26.3 ± 5.4	0.600
	Body mass index	24.6 ± 1.8	24.9 ± 1.4	0.438
	Seasons in NBA prior to injury	5.3 ± 3.1	5.3 ± 3.2	0.969
Injury setting				
	Non-basketball related	1 (3.0)		
	Playoff game	4 (12.1)		
	Practice	1 (3.0)		
	Preseason game	2 (6.1)		
	Preseason practice	1 (3.0)		
	Preseason training camp	1 (3.0)		
	Preseason workout	1 (3.0)		
	Regular season game	17 (51.5)		
	Regular season practice	3 (9.1)		
	Unable to confirm	2 (6.1)		
	Total	33 (100)		

The average time from injury to surgery was 3.3 ± 4.9 weeks. Thirteen (36.4%, 13/33) thumb collateral ligament surgeries occurred in the off-season or were season-ending. Twenty (60.6%, 20/33) players returned to sport in the same season as their surgery. RTS time between thumb RCL and UCL was comparable (Table 2). Twenty-one (63.64%, 21/33) players had thumb collateral ligament surgery on their dominant hand/thumb and 12 (36.4%, 12/33) players had surgery on their non-dominant hand/thumb. There was no difference in career length for the case group compared to the control group when evaluating career length (case: 9.6 ± 4.1, control: 9.4 ± 4.3, p > 0.001) and games per season (case: 44.9 ± 14.8, control: 53 ± 14.2, p > 0.001).

**Table 2:** Return to sport (RTS) timing after thumb collateral ligament surgery * RCL= radial collateral ligament; UCL= ulnar collateral ligament. ** Presented as mean ± SD.

	In-season injury				Off-season injury			
	Overall (n = 20)	Thumb RCL (n = 4)	Thumb UCL (n = 7)	Unspecified (n = 9)	Overall (n = 13)	Thumb RCL (n = 3)	Thumb UCL (n = 3)	Unspecified (n = 7)
RTS time (weeks)	7.1 ± 2.4	6.9 ± 1.3	7.5 ± 2.2	6.9 ± 2.2	28.4 ± 18.7	19.4 ± 8.0	27.0 ± 5.9	33.0 ± 24.5

Thumb UCL (58.8%, 10/17) and RCL (41.2%, 7/17) were 51.5% (17/33) of specified thumb collateral ligament surgeries. The specified ligament (UCL and RCL) had no significant association (p > 0.001) with career length or games per season (Table 3).

**Table 3:** Career length and post-index games per season, stratified by operative thumb collateral ligament * RCL= radial collateral ligament; UCL= ulnar collateral ligament. ** Statistics presented as mean ± SD.

	Thumb RCL (n = 7)			Thumb UCL (n = 10)			Unspecified (n = 16)		
	Case	Control	p-value	Case	Control	p-value	Case	Control	p-value
Career length	14.1 ± 2.5	13.3 ± 2.6	0.48	9.1 ± 4.2	8.8 ± 4.3	0.864	10.4 ± 4.5	9.7 ± 4.2	0.635
Games/season	47.3 ± 15.4	58.5 ± 13.2	0.168	47.2 ± 18.1	54.9 ± 12.1	0.28	47.8 ± 12.7	48.5 ± 13.5	0.888

The remaining 48.5% (16/33) did not identify which thumb collateral ligament was operated on. The laterality (left or right) of the operative collateral ligament had no significant association (p > 0.001) with career length or games per season. Surgery on the UCL or RCL was not significantly associated (p > 0.001) with pre-index or post-index performance when compared to the control group.

**Figure 3:**
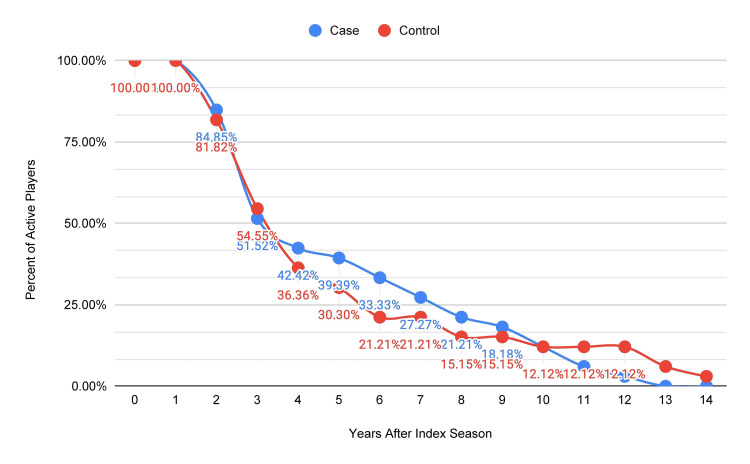
Kaplan-Meier career survival curve comparing case group to control group (n = 33, both groups) * Year 0 is pre-index.

The five-year NBA career survival rate of players undergoing thumb collateral ligament surgery was 39.4% (13/33), which was greater than the control group (27.3%, 9/33) (Figure 3). The control group's career length (4.9 ± 3.7) was not significantly different from the case group (5.5 ± 3.5, p = 0.910).

A statistically significant decrease in games per season (pre-index = 65.9 ± 12.1, post-index = 52.6 ± 13.5, p < 0.001) was identified between pre-index performance and post-index performance in the control group (Table 4). Similarly, a statistically significant decrease in games per season was identified in the case group (pre-index = 64.7 ± 10.1, post-index = 47.5 ± 14.6, p < 0.001) (Table 5). There were no significant differences (p > 0.001) between case group pre-index performance statistics and control group pre-index performance (Table 6). There were no significant differences (p > 0.001) between the case group's post-index performance statistics and the control group's post-index performance statistics (Table 7).

**Table 4:** Comparison of pre-index performance to post-index performance in the control group * Categories presented relative to the date of surgery (index date). ** Statistics presented as mean ± SD. *** A significant decrease in games/season was also observed in the case group.

Performance statistics		Pre-index	Post-index	p-value
	Seasons	5.3 ± 3.2	4.9 ± 3.7	0.643
	Games/season	65.9 ± 12.1	52.6 ± 1 3.5	<0.001
	Minutes per game	26.9 ± 6.8	26.2 ± 6.5	0.654
	Field goal percentage	46.0 ± 5.1	45.2 ± 6.3	0.663
	Three-point percentage	29.9 ± 11.3	32.3 ± 10.3	0.376
	Two-point percentage	49.1 ± 4.7	49.3 ± 5.6	0.857
	Effective field goal percentage	50.3 ± 3.6	51.6 ± 4.9	0.198
	Free throw percentage	76.7 ± 7.4	77.3 ± 8.1	0.778
	Points per game	11.8 ± 4.2	11.5 ± 5.2	0.82
	Total rebounds per game	4.6 ± 2.4	4.5 ± 2.6	0.799
	Assists per game	2.8 ± 2.1	2.8 ± 1.7	0.958
	Steals per game	0.9 ± 0.3	0.8 ± 0.3	0.363
	Blocks per game	0.5 ± 0.6	0.5 ± 0.6	0.954
	Turnovers per game	1.6 ± 0.7	1.5 ± 0.6	0.352
	Personal fouls per game	2.3 ± 0.6	2.1 ± 0.5	0.083
League-adjusted shooting				
	Field goal+	100.8 ± 10.9	99.3 ± 13.7	0.617
	Two-point shooting+	100.3 ± 9.1	98.2 ± 10.1	0.379
	Three-point shooting+	89.7 ± 23.7	96.2 ± 16.5	0.223
	Effective field goal percentage+	100.6 ± 7.3	101.0 ± 8.6	0.855
	Free throw percentage+	101.2 ± 10.1	101.3 ± 10.7	0.970
	True shooting percentage+	100.5 ± 6.5	100.2 ± 7.8	0.882
	Free throw attempt rate+	101.1 ± 44.9	90.8 ± 45.8	0.366
	Three-point attempt rate+	98.6 ± 69.3	122.4 ± 74.0	0.193

**Table 5:** Case group comparison of pre-index performance to post-index performance in National Basketball Association (NBA) athletes undergoing thumb collateral ligament surgery * Categories presented relative to the date of surgery (index date). ** Statistics presented as mean ± SD. *** A significant decrease in games/season was also observed in the control group.

Performance statistics		Pre-index	Post-index	p-value
	Seasons	5.3 ± 3.1	5.5 ± 3.5	0.882
	Games/season	64.7 ± 10.1	47.5 ± 14.6	<0.001
	Minutes per game	26.8 ± 6.9	25.6 ± 6.7	0.471
	Field goal percentage	45.4 ± 5.8	45.3 ± 6.1	0.946
	Three-point percentage	27.3 ± 13.3	29.6 ± 12.9	0.487
	Two-point percentage	47.9 ± 5.6	48.5 ± 5.3	0.682
	Effective field goal percentage	49.4 ± 4.0	50.9 ± 4.3	0.161
	Free throw percentage	75.5 ± 9.5	75.9 ± 10.5	0.881
	Points per game	11.9 ± 5.03	11.3 ± 5.0	0.621
	Total rebounds per game	4.4 ± 2.3	4.5 ± 2.7	0.958
	Assists per game	3.0 ± 2.2	2.8 ± 2.1	0.736
	Steals per game	1.1 ± 0.5	0.9 ± 0.4	0.191
	Blocks per game	0.6 ± 0.7	0.6 ± 0.6	0.983
	Turnovers per game	1.7 ± 0.7	1.4 ± 0.6	0.189
	Personal fouls per game	2.4 ± 0.5	2.2 ± 0.6	0.255
League-adjusted shooting				
	Field goal+	99.5 ± 12.5	99.1 ± 13.3	0.887
	Two-point shooting+	98.1 ± 11.9	96.4 ± 10.0	0.526
	Three-point shooting+	90.7 ± 18.8	94.5 ± 18.8	0.454
	Effective field goal percentage+	99.1 ± 8.6	99.4 ± 7.9	0.882
	Free throw percentage+	99.6 ± 12.5	99.4 ± 12.6	0.955
	True shooting percentage+	99.4 ± 7.8	99.1 ± 7.3	0.859
	Free throw attempt rate+	110.5 ± 34.1	99.4 ± 38.5	0.217
	Three-point attempt rate+	97.3 ± 67.2	108.2 ± 73.7	0.54

**Table 6:** Pre-index performance comparison between the case group and the control group * Statistics presented as mean ± SD.

Performance statistics		Case	Control	p-value
	Seasons	5.3 ± 3.1	5.3 ± 3.2	0.969
	Games/season	64.7 ± 10.1	65.9 ± 12.1	0.660
	Minutes per game	26.8 ± 6.9	26.9 ± 6.8	0.931
	Field goal percentage	45.4 ± 5.8	46.0 ± 5.1	0.616
	Three-point percentage	27.3 ± 13.3	29.9 ± 11.3	0.397
	Two-point percentage	47.9 ± 5.6	49.1 ± 4.7	0.358
	Effective field goal percentage	49.4 ± 4.0	50.3 ± 3.6	0.372
	Free throw percentage	75.5 ± 9.5	76.7 ± 7.4	0.573
	Points per game	11.9 ± 5.03	11.8 ± 4.2	0.909
	Total rebounds per game	4.4 ± 2.3	4.6 ± 2.4	0.754
	Assists per game	3.0 ± 2.2	2.8 ± 2.1	0.659
	Steals per game	1.1 ± 0.5	0.9 ± 0.3	0.078
	Blocks per game	0.6 ± 0.7	0.5 ± 0.6	0.593
	Turnovers per game	1.7 ± 0.7	1.6 ± 0.7	0.911
	Personal fouls per game	2.4 ± 0.5	2.3 ± 0.6	0.697
League-adjusted shooting				
	Field goal+	99.5 ± 12.5	100.8 ± 10.9	0.653
	Two-point shooting+	98.1 ± 11.9	100.3 ± 9.1	0.414
	Three-point shooting+	90.7 ± 18.8	89.7 ± 23.7	0.863
	Effective field goal percentage+	99.1 ± 8.6	100.6 ± 7.3	0.442
	Free throw percentage+	99.6 ± 12.5	101.2 ± 10.1	0.574
	True shooting percentage+	99.4 ± 7.8	100.5 ± 6.5	0.551
	Free throw attempt rate+	110.5 ± 34.1	101.1 ± 44.9	0.339
	Three-point attempt rate+	97.3 ± 67.2	98.6 ± 69.3	0.939

**Table 7:** Post-index performance comparison between the case group and the control group * Statistics presented as mean ± SD.

Performance statistics		Case	Control	p-value
	Seasons	5.5 ± 3.5	4.9 ± 3.7	0.515
	Games/season	47.5 ± 14.6	52.6 ± 13.5	0.148
	Minutes per game	25.6 ± 6.7	26.2 ± 6.5	0.632
	Field goal percentage	45.3 ± 6.1	45.2 ± 6.3	0.919
	Three-point percentage	29.6 ± 12.9	32.3 ± 10.3	0.340
	Two-point percentage	48.5 ± 5.3	49.3 ± 5.6	0.499
	Effective field goal percentage	50.9 ± 0.04	51.6 ± 4.9	0.511
	Free throw percentage	75.9 ± 10.5	77.3 ± 8.1	0.629
	Points per game	11.3 ± 5.0	11.5 ± 5.2	0.809
	Total rebounds per game	4.5 ± 2.7	4.5 ± 2.6	0.920
	Assists per game	2.8 ± 2.1	2.8 ± 1.7	0.988
	Steals per game	0.9 ± 0.4	0.8 ± 0.3	0.224
	Blocks per game	0.6 ± 0.6	0.5 ± 0.6	0.553
	Turnovers per game	1.4 ± 0.6	1.5 ± 0.6	0.666
	Personal fouls per game	2.2 ± 0.6	2.1 ± 0.5	0.329
League-adjusted shooting				
	Field goal+	99.1 ± 13.3	99.3 ± 13.7	0.956
	Two-point shooting+	96.4 ± 10.0	98.2 ± 10.1	0.476
	Three-point shooting+	94.5 ± 18.8	96.2 ± 16.5	0.722
	Effective field goal percentage+	99.4 ± 7.9	101.0 ± 8.6	0.484
	Free throw percentage+	99.4 ± 13.7	101.3 ± 10.7	0.620
	True shooting percentage+	99.1 ± 7.3	100.2 ± 7.8	0.603
	Free throw attempt rate+	99.4 ± 38.5	90.8 ± 45.8	0.437
	Three-point attempt rate+	108.2 ± 73.7	122.4 ± 74.0	0.484

## Discussion

The presented study determined surgery involving the thumb RCL or UCL does not significantly impact career length or performance when compared to matched controls. Most players underwent surgery on their dominant hand/thumb, but no differences were identified when comparing career length or performance for the dominant or non-dominant hand/thumb groups. The study hypotheses were supported by the investigation with a 100% RTS rate for all confirmed ligament surgeries with one year of preoperative and postoperative statistics, comparable career lengths, and no significant differences in performance when compared to controls or their own preoperative performance. Both groups played significantly less games per season after the index date representing surgery, but no between-group differences were identified. The 100% RTS rate would have also been identified had we not applied our inclusion criteria for the RTS rate and performance analysis. In other words, all 47 players with operatively managed thumbs continued playing professional basketball after their surgery for at least a year, even if not in the NBA.

Taylor et al. 2013 [14] reported a retrospective chart review of 56 patients from a military institution and identified rates of thumb MCP joint surgery at 48% overall, 67% for thumb UCL, and 40% for thumb RCL. Stoop et al. [15] reported a chart review of 383 patients from three urban hospitals with thumb UCL injuries and identified an overall surgery rate of 33%, which increased to 44% when evaluating men in their study. The presented study identified an MCP joint collateral ligament injury rate of 73.4%. This could be due to a lack of injury reports in the form of press releases for thumb MCP joint collateral ligament injuries when managed non-operatively.

RTS rate after thumb UCL surgery was determined by a similar methodology in Major League Baseball (MLB) and National Football League (NFL) athletes and had a similarly high rate (>95% in both studies) [4,16]. The time to RTS in the presented study (7.5 ± 2.2 weeks) was also comparable to MLB (eight weeks), but NFL athletes did not return to sport for an average of almost 19 weeks. Neither study identified significant differences in postoperative performance when compared to controls [4,16]. The longer time to RTS identified in NFL athletes compared to the presented study may be attributable to NFL athletes delaying operative treatment of isolated thumb UCL tears until the offseason, leading to a longer time to RTS number that is not indicative of their RTS status (i.e., these athletes return to off-season training or pre-season games) [16,17]. Gibbs et al. 2020 [6] described RTS time in 18 primary thumb UCL repairs (reconstruction excluded) of athletes in multiple sports. Their timing for in-season UCL repair (4.4 weeks) was quicker than the presented study (7.5 weeks), but RTS was across multiple sports and may contribute to the reported differences. Additionally, management options for isolated UCL tears vary amongst hand surgeons with options ranging from immediate postoperative RTS to RTS up to three months postoperatively [18]. For example, postoperative bracing and return to play protocols utilizing a brace may vary from surgeon to surgeon and our study was not able to identify if return to sport times were affected by the presence or absence of a brace. This is likely dependent on an individual surgeon's preference, which this study could not evaluate.

The authors were unable to identify a previous study evaluating the RCL in high-level athletes playing basketball with outcomes including RTS rate, time to RTS, and evaluation of postoperative performance. Werner et al. (2017) [17] described simultaneous RCL/UCL tears in their case series with NFL athletes. Five thumbs were simultaneous RCL/UCL tears in-season that delayed surgery to the off-season. Four athletes with simultaneous RCL/UCL tears had the injury occur in the off-season and had off-season surgery. They reported a 100% RTS rate without limitation in the season following surgery. The high-energy injury mechanism leading to simultaneous collateral ligament rupture may be unique to NFL athletes, precluding this study from comparison. No other studies evaluating isolated RCL injury in high-level athletes (collegiate, professional) were identified by the authors. Catalano et al. [19] reported a chart review of 26 patients (military population) with operatively managed RCL tears (16 repairs and 10 reconstructions) and one-year follow-up. They reported no significant differences in MCP stability, MCP/interphalangeal joint motion, and grip/pinch strength for either method of repair. Coyle (2003) [20] reported on 45 patients with grade III RCL injuries with outcomes including full range of motion of the MCP joint (79%), asymptomatic (87%), and normal pinch/grip strength (95%). Both studies concluded grade III RCL injuries can be operatively managed successfully [19,20].

Both groups (case and control) in the presented study had a significant decrease in games per season when comparing pre-index to post-index with no between-group difference. Considering these findings, it is most likely that this is part of an NBA athlete's natural career trajectory rather than a result of a thumb collateral ligament injury. However, this study cannot infer causality for the identified finding and this would require its own separate investigation. No identifiable difference between dominant/non-dominant thumb injuries may be reassuring for NBA athletes, as the possibility of a dominant thumb injury being more severe does not appear to be true for this specific injury.

This observational study is not without limitations. The use of a search strategy may have caused some players to go unidentified by the search or be missed during the article review. Additionally, the publicly available information did not specify the operative ligament in almost half of the reports. No reports identified simultaneous UCL/RCL injuries. The data collected did not include information on repair or reconstruction. The presented study did not utilize medical records but relied on media reports. Therefore, the accuracy of the presented information is unable to be verified. Some, but not all, press releases provide information about who the operating surgeon was and their affiliation. This information was not collected and could be relevant information to the presented study. No information is available from the athlete’s experiences with thumb collateral ligament surgery (patient-reported outcomes, functional scores at follow-ups). This information could have been utilized to compare non-operative to operative treatment and subsequent outcomes. There is a risk of this study being underpowered when subgroup analysis by collateral ligament injury (RCL vs. UCL vs. unidentified) was performed due to the small sample sizes. While time to RTS varies, the presented study utilized the most commonly reported methodology [21].

## Conclusions

The presented study identified a high rate of RTS in NBA athletes undergoing thumb UCL and RCL surgery. Players do not experience decreased performance or career length due to thumb collateral ligament surgery, regardless of a dominant or non-dominant thumb injury. RTS and performance are not impacted by thumb MCP joint UCL or RCL injuries in NBA athletes.
